# Supplementary material for: Light sheet microscopy with acoustic sample confinement
Source: Nat Commun. 2019 Feb 8;10:669. doi: 10.1038/s41467-019-08514-5 (PMC6368588; doi:10.1038/s41467-019-08514-5)
Supplement: Supplementary file 1 — Supplementary Information [file 41467_2019_8514_MOESM1_ESM.pdf]

Supplementary Materials for

# Light sheet microscopy with acoustic sample confinement

Yang et al.

# Supplementary Note 1: Diffusion speed of fluorescein in water and agarose

The speed of molecular diffusion depends on parameters such as temperature, viscosity of the fluid and the mass of the compound. To demonstrate the different diffusion speeds of a compound in water and agarose, we investigated fluorescein's diffusion process since its concentration can be easily quantified with the fluorescent signal intensity.

First, we tested the delivery of fluorescein solution into the sample chamber with our circulation system. To be more specific, purified water was circulated in the sample chamber and a beaker with PVC tubing, with the total volume of water to be 200ml. Before the start of the measurement, the peristaltic pump was stopped, then 2ml of 0.1 mM fluorescein was added into the beaker. The 488nm laser was delivered to the center of the sample chamber, and the fluorescent signal of fluorescein was collected with the existing LSFM, at the same time as the pump was started. Since the intensity of the signal is linearly proportional to the concentration of fluorescein, this serves well for quantifying the relative concentration of fluorescein at the imaging point. The fluorescent signal was recorded every 2 seconds for 30 minutes.

This was repeated with an agarose cylinder suspended in the center of the sample chamber. The illumination laser was delivered to the center of the agarose rod whilst the fluorescent signal was recorded. All the other conditions were the same. The test was repeated with 1mm diameter and 2mm diameter agarose rods.

Fluorescent intensity integrated across the whole frame is plotted below:

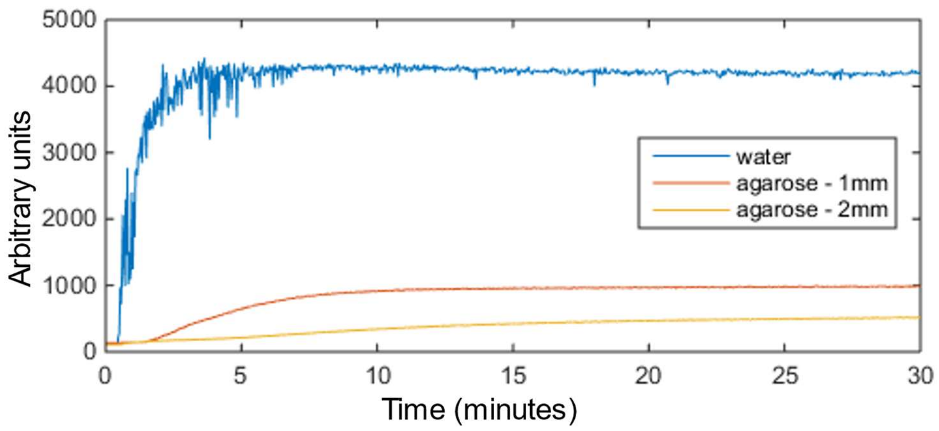


*Supplementary Figure 1: Fluorescein diffusion test for water and agarose rod.*

Without agarose, it took less than 1 minute for the compound to reach the imaging area. Then the concentration of the compound reached the top at around three minutes. Whilst in agarose, the diffusion is much slower. After 30 minutes, the fluorescein concentration in agarose is only approximately a quarter of that in water.

## Supplementary Note 2: Supplemental drug study utilizing this system

Additional experiments were carried out on older 3-dpf zebrafish to validate the effect of age on heart rate increase. Supplementary Figure 2 shows a significantly higher response to norepinephrine than observed in 2-dpf zebrafish, shown in the main text. Developmental age has profound influence on the capacity of the cardiovascular system to respond to drugs [1], which is reflected here. The mean increase of approximately 17% is consistent with the previous literature [2].

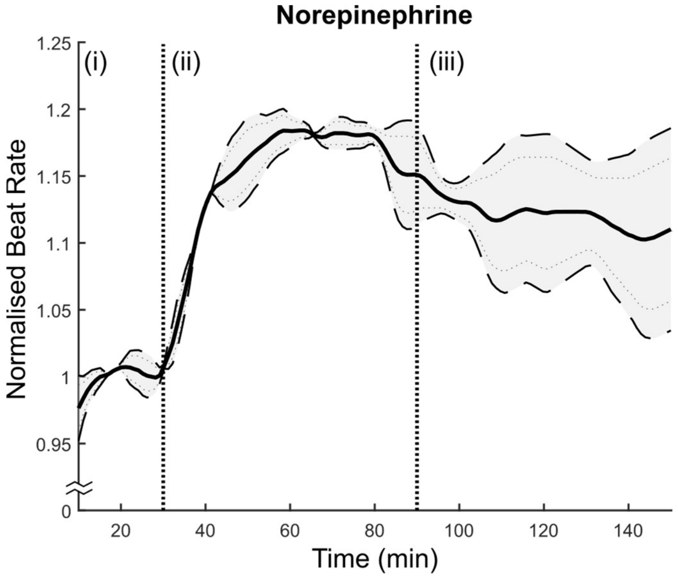


*Supplementary Figure 2: Normalised heart rate in 3 days-past-fertilisation zebrafish with the addition of 1 mM of norepinephrine. No drug was added in period (i) for 30 minutes, drug was added in period (ii), and washed out in period (iii). The plot is presented as a mean value (solid lines) with error bounds of one standard deviation (dashed lines) across all results (dotted lines; n=2).*

In addition to the focused drug treatment described in the main text of our paper, further tests were conducted with *older* zebrafish larvae, as shown in Supplementary Figure 3. Verapamil and high-dose tricaine were given, respectively, to 5-day-old and 4-day-old zebrafish, following the same procedure as in the manuscript.

Due to the greater age of these zebrafish, their transparency was reduced, leading to reduced image quality. Nevertheless, we were able to extract the heart rate via video analysis.

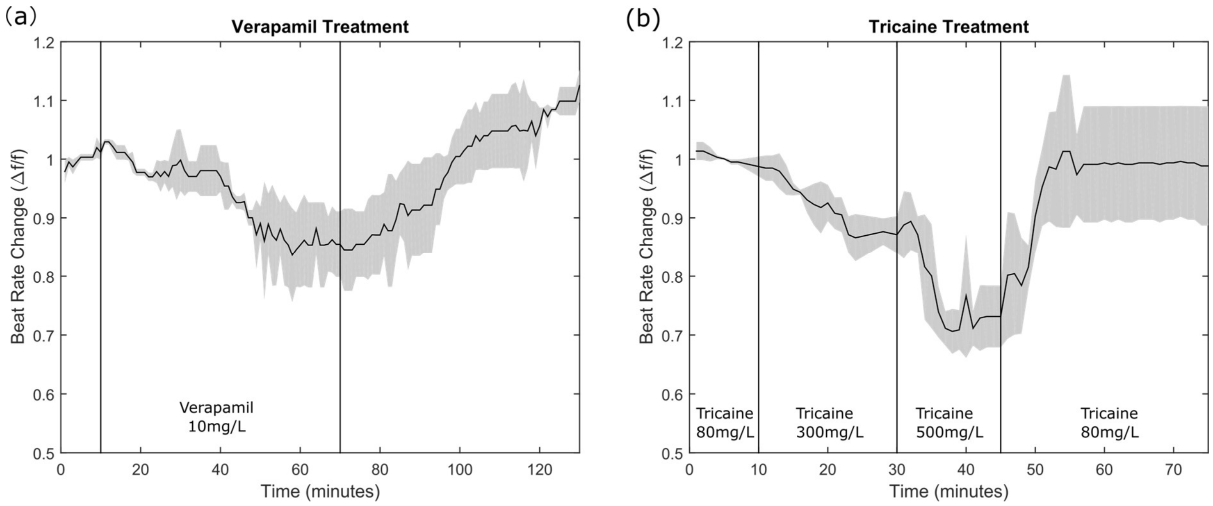


*Supplementary Figure 3: Drug treatment for older zebrafish larvae. Both graphs were acquired with three different samples. (a) Verapamil treatment for 5-day-old zebrafish larvae. The first 10 minutes of the recording was with baseline tricaine levels of 80mg/L. Verapamil was added after 10 minutes, resulting in a final concentration of 10 mg/L. After an additional hour of monitoring, fresh E3 medium (with 80mg/L tricaine) was added to wash away the Verapamil. Over the course of this experiment, a statistically significant drop and rise in heartbeat rate were observed. (b) Tricaine treatment. First 10 minutes was with 80mg/L tricaine. High-dose tricaine was added after 10 minutes, resulting in a final concentration of 300mg/L. Then a further high dose was added, resulting in 500mg/L. At the final stage, baseline tricaine was added to wash the high dose away.*

# Supplementary Note 3: Optical performance of the ETL

The electrically tunable lens (ETL) is a liquid lens that provides tunability over a focal power range from 5 dioptres (200 mm) to 10 dioptres (100 mm). This focal power range is offset by -10 dioptres *via* addition of a concave lens with f = -100 mm at the back of ETL. The position of the image plane is changed by applying a current to the ETL, ranging from 0 to 292 mA. With the present arrangement, an infinite focus of the ETL was achieved with a driving current 220mA. For most of our work, the ETL was driven with a triangular waveform, providing a range from 170 to 270mA.

**Scanning depth range**

The scan depth of the ETL was calibrated using a low frequency (10 Hz) drive waveform. The measurement was performed by manually translating a fluorescent bead along the detection axis and monitoring the range over which the bead appeared / disappeared from view. In the arrangement shown in Supplementary Figure 6a, the manual stage provided translation along a direction that is diagonally disposed with respect to the detection path, which just introduces a factor of $\sqrt{2}$ into the calibration. Upon repeating the above procedure six times and averaging the results, we obtained the scan range of the ETL driven at 170 to 270mA as 249.6 ± 3.0 µm. This range can be extended by driving the ETL with a larger amplitude waveform.

**Optical resolution**

The ETL is meant to reside in the back focal plane of the objective lens, which could be achieved *via* projection optics. However, in our compact setup, we simply place the ETL directly after the objective, where it is oriented at 45° with respect to vertical. As the ETL membrane is elastic, the lens shape is influenced by gravity. Such issues demand that optical resolution be experimentally assessed for this arrangement.

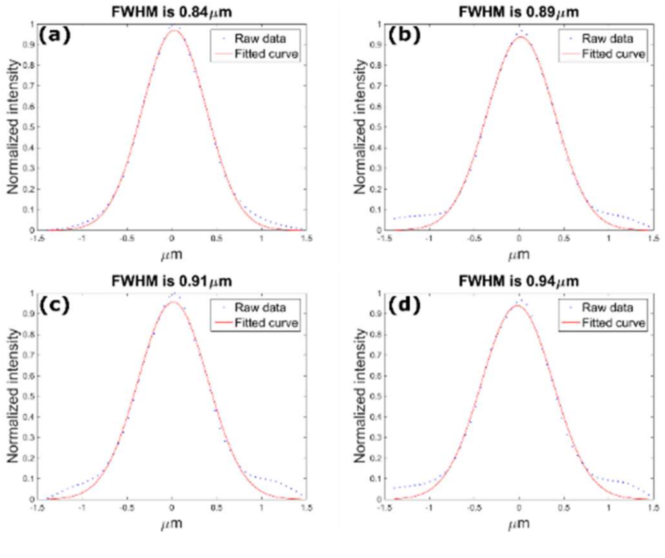


*Supplementary Figure 4: Resolution characterization, showing the resolution (a) without ETL, (b) with ETL run at 220 mA, (c) with ETL run at 170 mA, (d) with ETL run at 270 mA.*

With a detection NA of 0.5 and the spectrum of the fluorescent signal centred on 0.6 µm, the diffraction-limited resolution ($0.61\lambda / NA$) is 0.73 µm. Without the ETL, the estimated resolution (based on FWHM of fitted Gaussian curve) is 0.84 µm (Supplementary Figure 4 (a)), which is slightly worse than the diffraction limit, potentially because the tube lens we used is not the optimal lens for the Olympus objective.

With the ETL introduced into the setup, optical resolution on three planes was examined, with a driving current on the ELT, Supplementary Figure 4(b) 170mA, (c) 220 mA and (d) 270 mA respectively. It can be seen that with the presence of the ETL, the contrast and resolution of the image are slightly affected, potentially because of the sub-optimal orientation of the ETL. With the ETL, we can still achieve submicron resolution.

To eliminate the effect of gravity, we have suggested an alternative optical setup, featuring dual-side illumination and a horizontally arranged ETL. Details are given in Supplementary Note 4: Alternative optical setup.

**Actual magnification**

Having the ETL directly after the objective, in contrast to a 4f system, leads to a change in the FOV when changing the focal length of the ETL [3]. We measured the resulting FOV by translating a reference target (a fluorescent bead) across the entire FOV and recording the reading on a manual actuator. This process was repeated three times and averaged to obtain the FOV, then repeated in three planes, i.e. ETL driven at 170 mA, 220 mA and 270 mA statically. The results are as follows:

*Supplementary Table 1: FOV evaluation of ETL different driving currents*

| Driving current (mA) | 170 | 220 | 270 |
|---|---|---|---|
| FOV (µm) | 546 | 580 | 617 |
| Magnification | 24.38 | 22.95 | 21.58 |
| Resolution (µm) | 0.91 | 0.89 | 0.94 |

We found that within this scanning range, the actual magnification difference is within ±6% and considered negligible. If necessary, this effect could be avoided by adding a 4f-system in the detection arm and placing the ETL between the two lenses of this system.

## Supplementary Note 4: Alternative optical setup

Here we discuss the potential merits of an alternative arrangement, shown in Supplementary Figure 5. This setup utilizes both sides of a *horizontally* oriented breadboard, with illumination path and sample chamber above the breadboard, while detection optics and the camera reside beneath the breadboard. This arrangement has several advantages:

1. This orientation of the ETL will minimize wavefront error due to the influence of gravity.
2. Specimen loading is simplified in this geometry: an acoustically trapped sample will automatically be in focus optically.
3. Dual-side illumination helps to improve image quality.

Otherwise, there is no fundamental difference between this arrangement and our current setup, so we believe such a conversion to be straightforward.

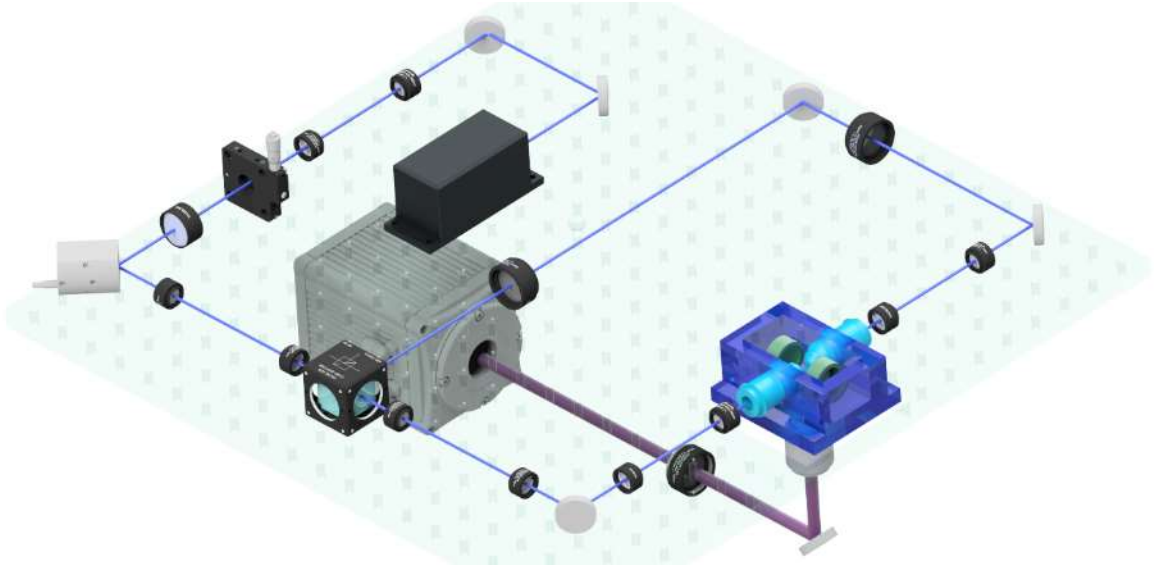

*Supplementary Figure 5: Alternative optical arrangement, featuring dual-side illumination, optimized orientation for ETL, and simplified sample loading. Again, a laser beam is expanded such that a light sheet is formed on a scanning mirror. Here, though, after going through a relay lens pair, the beam is split into two by a 50:50 beam splitter. The relay lenses on two arms have the same ratio, so the result scanning light sheets coincide in the sample chamber. This compact setup only occupies a space of 50 × 50 × 20 cm$^3$.*

# Supplementary Note 5: Experimental setup

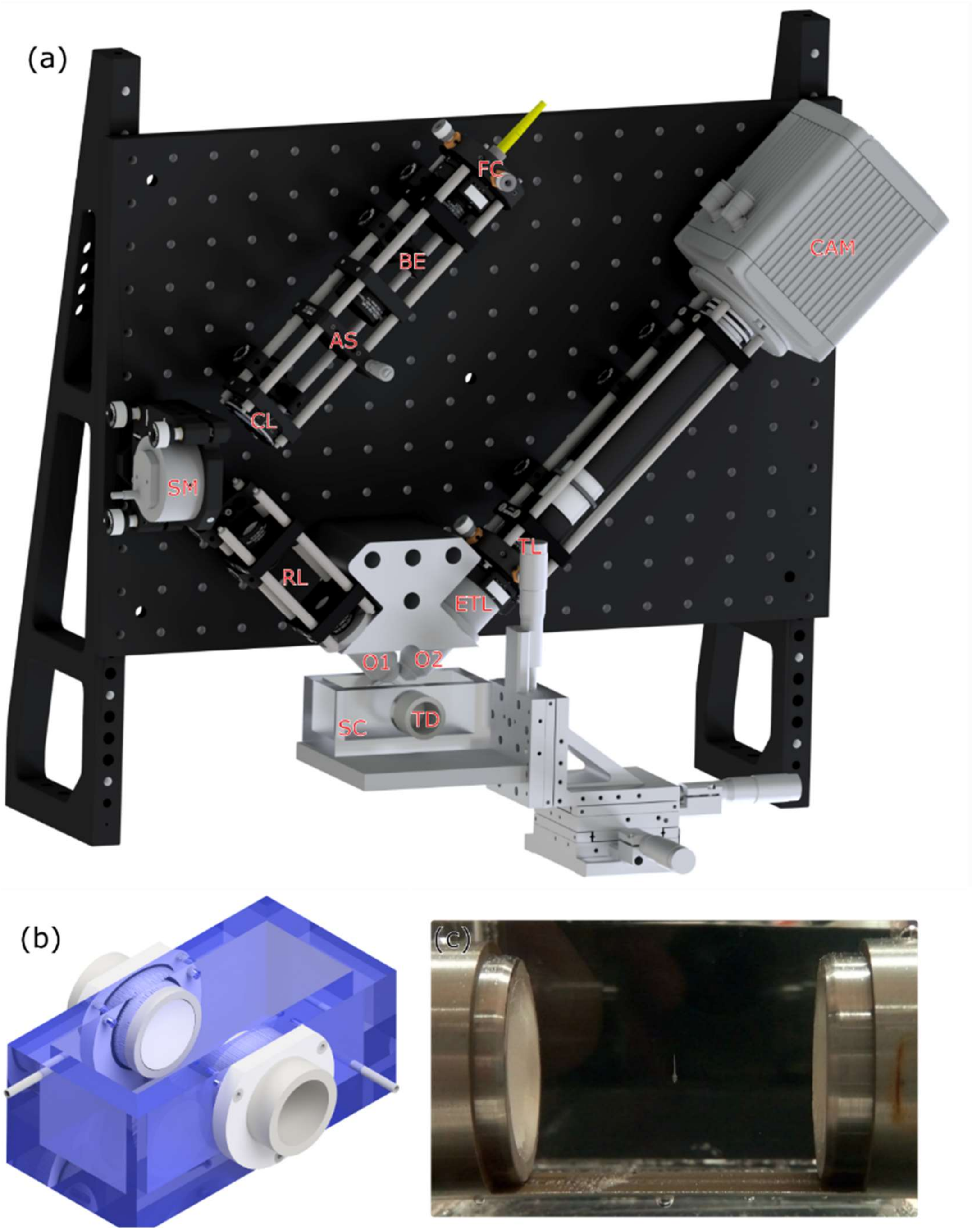


*Supplementary Figure 6: Light sheet setup with the sample chamber. (a) Laser beam is introduced by a single-mode fiber (FC), is expanded (BE), then goes through an adjustable slit (AS) and cylindrical lens (CL). A a pair of relay lenses (RL) transmits the sheet to the entrance pupil of the illumination objective (O1). The detection objective (O2) projects images onto a sCMOS camera (CAM). A piezo scanning mirror (SM) and electrically tunable lens (ETL) simultaneously shift the light sheet and detection plane, so focus is maintained whilst 3D image stacks are accumulated on a stationary sample, which is acoustically trapped by transducers (TD) in a 80 × 30 × 30 mm$^3$ sample chamber (SC)) filled with a medium which is optimal for the sample. A manual translation stage is used only to place the chamber into the imaging position. (b) 3D rendering of the sample chamber with acoustic transdcuers. (c) Photomicrograph of the chamber with acoustically trapped zebrafish.*

# Supplementary Note 6: Hardware control between multiple devices.

Synchronization of the scanning mirror (SM) and electrically tunable lens (ETL) shown in Supplementary Figure 6 is critical to maintaining well-matched light sheet position and detection plane. This requires precise control of the amplitude and phase of displacements of both devices. Supplementary Figure 7 illustrates our method: the ETL controller (Electrical Lens Driver 4, Optotune) runs as a master device. It outputs a triggering signal during each scan cycle, which is passed to a two-channel function generator (AFG1022, Tektronix), set up to use this trigger (in burst mode) to control the phase of the scanning mirror. All hardware is controlled through MATLAB software so other parameters such as the frequency and amplitude of the scanning mirror may either be changed manually or through the desktop control.

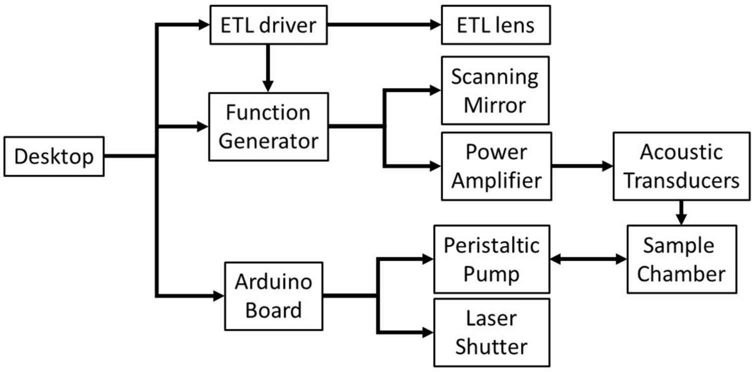


*Supplementary Figure 7: Diagram of the hardware control*

The second channel of the function generator outputs a sinusoid at the resonant frequency of the ultrasonic transducers, which is amplified to the required levels by a bespoke RF power amplifier.

Additional hardware includes a peristaltic pump (MINIPULS 3, Gilson) used to circulate fluid in the sample chamber and a laser shutter. Both the pump and the laser shutter were controlled remotely, *via* serial port communication with an Arduino board.

# Supplementary Note 7: Ultrasonic transducer / resonant device design and modeling

To design the ultrasonic force control system, a spherically concave, single-element transducer and a pair of confocal transducers were modeled using a finite-element analysis (FEA) package (PZFlex Ltd, Glasgow, UK), allowing comparison of predicted behavior and experimental performance.

*Supplementary Table 2: Material properties assumed for FEA predictive models*

| Material | Water | Air | Epoxy | PZ26 |
|---|---|---|---|---|
| Density ($kg{\cdot}m^{-3}$) | 1000 | 1.24 | 1149 | 7700 |
| Speed of sound ($m{\cdot}s^{-1}$) | 1496 | 343 | 2536 | 4727 |

The symmetry of the experimental system suggested the use of two-dimensional (2D) axisymmetric computational models, for which we input the material properties listed in Supplementary Tables 2 and 3. In the PZFlex modeling environment, any given piezoelectric material can be assigned only one unique poling direction, and so our model of a radially poled, spherically concave single-element PZ26 transducer was divided into 88 arc sections, each subtending an angle of 0.879°, with individual poling directions directed towards the focal point. The FEA mesh size was $40 \times 40$ µm$^2$, which is approximately 4% of the wavelength in water at 1.5 MHz, providing a high degree of confidence in the results. In both models, the column of air behind the PZ26 components was represented as a free boundary condition assigned at the maximum of the *x*-axis. For the confocal device model, a symmetrical boundary condition was assigned at the minimum of the *x*-axis. An absorbing boundary condition was assigned at the other limits of the *x*-axis and at both limits of the *y*-axis for both models.

The single-element model was excited with a one-cycle sinusoid at 1.5 MHz and the simulation was allowed to run until oscillation decayed to 10% of its maximum. The model confocal system was excited with the same signal but runtime was adjusted to allow the ultrasonic pulse to complete eight one-way trips between the two transducers.

*Supplementary Table 3: Piezoelectric properties of PZ26: elastic stiffness constant, $c_{ij}^E$ ($10^{10}$ N/m$^2$), piezoelectric constant, $e_{ij}$ (C/m$^2$) and relative permittivity, $\varepsilon_{ij}^S$ ($\varepsilon_0$)*

| $c_{11}^E$ | $c_{12}^E$ | $c_{13}^E$ | $c_{33}^E$ | $c_{44}^E$ | $c_{66}^E$ | $e_{15}$ | $e_{31}$ | $e_{33}$ | $\varepsilon_{11}^S$ | $\varepsilon_{33}^S$ |
|---|---|---|---|---|---|---|---|---|---|---|
| 16.8 | 11.0 | 9.99 | 12.3 | 3.01 | 2.88 | 9.86 | -2.8 | 14.7 | 828 | 700 |

Electrical impedance spectra for each model were derived from the simulated voltage and current responses. Supplementary Figure 8 compares these simulated electrical impedance spectra with our experimentally measured results for one device geometry. The relatively good agreement was taken to support the use of such simulations for device design.

Supplementary Figure 9 shows our simulated results for a focused ultrasound beam from the single-element transducer at $f$ = 1.468 MHz, given an excitation amplitude of 1 $V_{peak}$. For the confocal system model, the pressure responses were recorded in the time domain at the geometric center of the water and at a point 1.2 µm away from the center on the $x$-axis. After performing a fast Fourier transform on the time-domain response and normalizing for the input voltage, the pressure response spectra were extracted as shown in Supplementary Figure 10. Because of the resonant nature of the system, the periodic system resonances vary compared with those of the ultrasonic transducers. As can be seen in Supplementary Figure 10, the resonances of the confocal system are superposed on the resonant behavior of the single element transducer. Additionally, the frequency of the maximum pressure response in the confocal system, $f$ = 1.519 MHz, is distinct from the resonant frequency of the single-element transducer.

The periodic system resonances shown are dependent upon the low level damping assumed for the resonance device and this is also linked to the fact that the wavelength of ultrasound in water is much less than the thickness of the water layer [4]. The resonant frequency of the transducer and the system resonant frequencies with peak pressure responses were selected to observe pressure distributions. Supplementary Figure 11(a) and (b) show the normalized ultrasound pressure fields of the confocal system driven at $f$ = 1.468 MHz and $f$ = 1.519 MHz, respectively. Significantly, the maximum pressure amplitude at $f$ = 1.519 MHz is *more than three times that* at $f$ = 1.468 MHz.

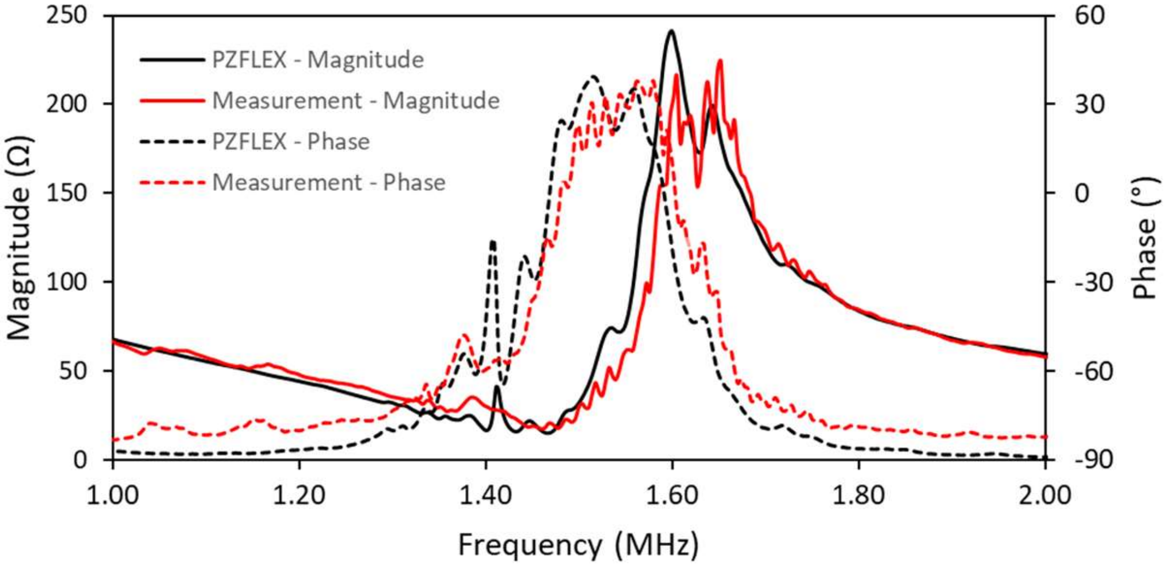


*Supplementary Figure 8: PZFlex simulated electrical impedance spectra of the spherically concave, single-element transducer compared with experimental measurement*

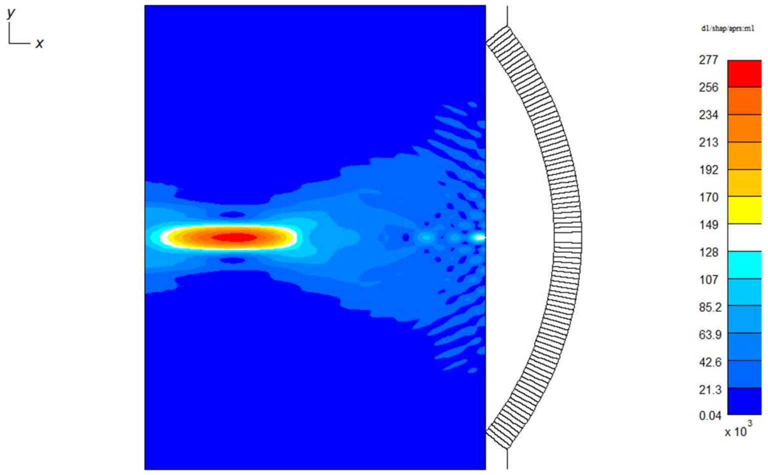


*Supplementary Figure 9: Simulated ultrasound pressure field of a single transducer driven at 1.468 MHz*

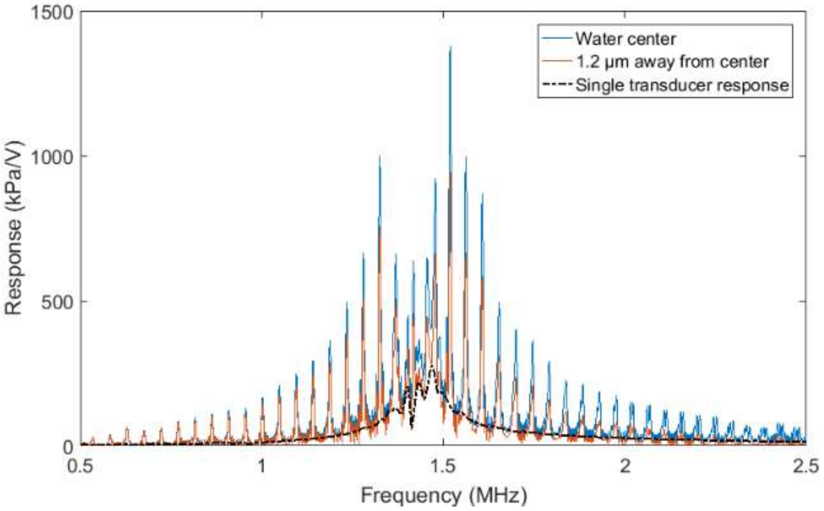


*Supplementary Figure 10: Pressure response spectra at the water center, and 1.2 μm away from the center, superposed on the resonance trend of the single transducer.*

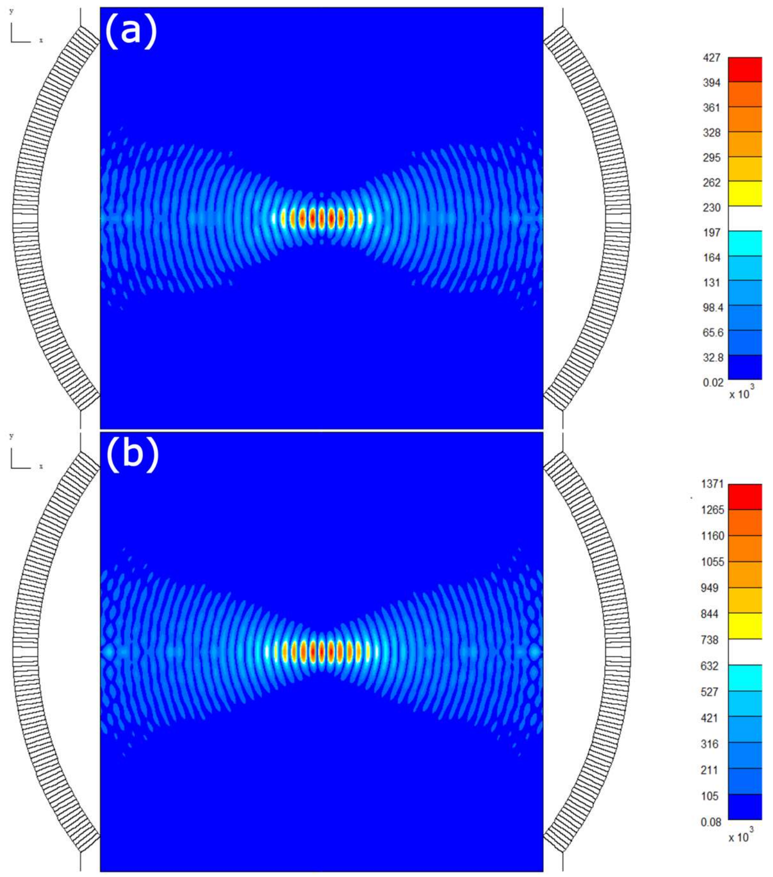


*Supplementary Figure 11: Simulated ultrasound pressure fields between two confocal ultrasound transducers driven at (a) 1.468 MHz and (b) 1.519 MHz.*

## Supplementary Note 8: Experimental characterization of ultrasound devices:

The electrical impedance magnitude and phase spectra of the fabricated transducers and the confocal system were experimentally measured using a specialized impedance analyzer (4395A, Agilent Technologies, CA, USA). The impedance of the confocal system was measured by connecting the two transducers in parallel. Impedance analyzer calibration and cable compensation were performed prior to each measurement. The default number of data points, $n = 801$, was taken over each frequency range of interest. The measurements were performed first on the transducers in air then on the system comprising the transducers coupled to the sample chamber filled with water. The resonant frequencies for Transducers 1 and 2 in air are approximately $f = 1.481$ MHz and $f = 1.478$ MHz with impedance magnitudes $|Z| = 6.2$ Ω and $|Z| = 6.1$ Ω, respectively. The system impedance magnitudes measured in water increase respectively to $|Z| = 17.9$ Ω and $|Z| = 13.6$ Ω because water provides higher acoustic damping than air. The impedance spectra shown in Supplementary Figure 12 include perturbations that are periodic in the frequency domain; as shown below, these can be definitively attributed to ultrasonic standing waves formed between the two transducers.

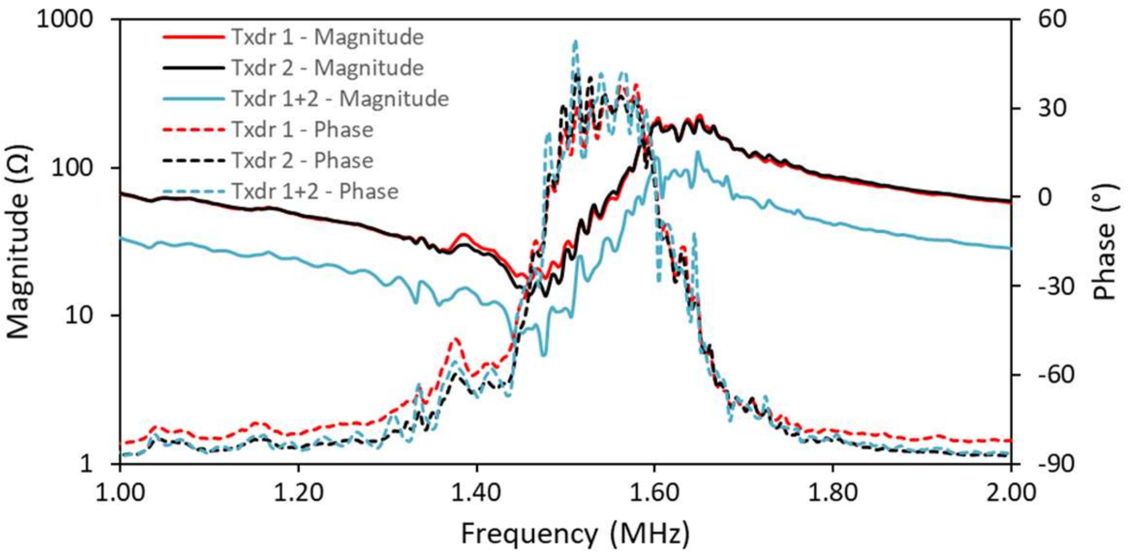


*Supplementary Figure 12: Electrical impedance spectra of two-transducer system measured for the confocal device with water-filled chamber.*

In order to directly visualize the ultrasonic standing waves formed in the water between the transducers, we performed field mapping experiments, schematically represented in Supplementary Figure 13. A calibrated needle hydrophone (Precision Acoustics Ltd, Dorset, UK) with a Ø 0.5 mm polyvinylidene fluoride (PVDF) sensor in a Ø 1.09 mm case was fixed on an *X-Y-Z* scanning stage. The hydrophone was first positioned close to the focal point. One transducer was excited with a 5 $V_{pp}$ continuous sinusoid by a signal generator with 50 Ω output resistance. By translating the hydrophone, we measured an ultrasonic standing wave formed by interference between the transmitted sound wave and its reflection from the front face of the other transducer. By manually sweeping the frequency across the range 1.45 MHz

< $f$ < 1.55 MHz with an increment, $\Delta f$ = 1 kHz, we found that the peak pressure amplitude was observed at $f$ = 1.495 MHz. Subsequently, the pressure field amplitude was mapped in space, using a 0.1 mm step size in the axial and lateral directions, with the transducer driven with $V$ = 5 $V_{pp}$ continuous sinusoid at $f$ = 1.495 MHz. The result is shown in Supplementary Figure 14(a). Further field mapping was performed with the two transducers in the confocal system connected electrically in parallel and driven simultaneously with the same signal. The maximum pressure amplitude of the confocal system was $P$ = 0.274 MPa as shown in Supplementary Figure 14(b).

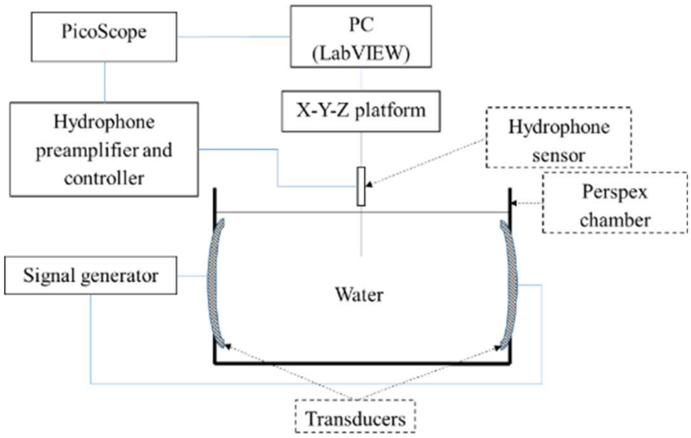


*Supplementary Figure 13: The experimental setup for ultrasound field mapping*

Assuming a linear system, the maximum acoustic pressure with 1V input in the confocal system is measured about 0.11 MPa at 1.495MHz. Hence, the maximum acoustic pressure about 0.82MPa was used for the cell experiments at 7.5V input. Despite this pressure was sufficient to trap all samples presented in the work, it is much smaller than the simulation results, i.e. 1.37MPa per 1V input at 1.519MHz and 0.427 MPa per 1V at 1.468MHz. The difference is mainly attributed to the uncertainty in the resonator fabrication, including the small variations of two ultrasound transducers and imperfect alignment during confocal system construction, and the electrical impedance mismatch between the confocal system (13Ω at 1.495MHz) and the driving electronics (50Ω).

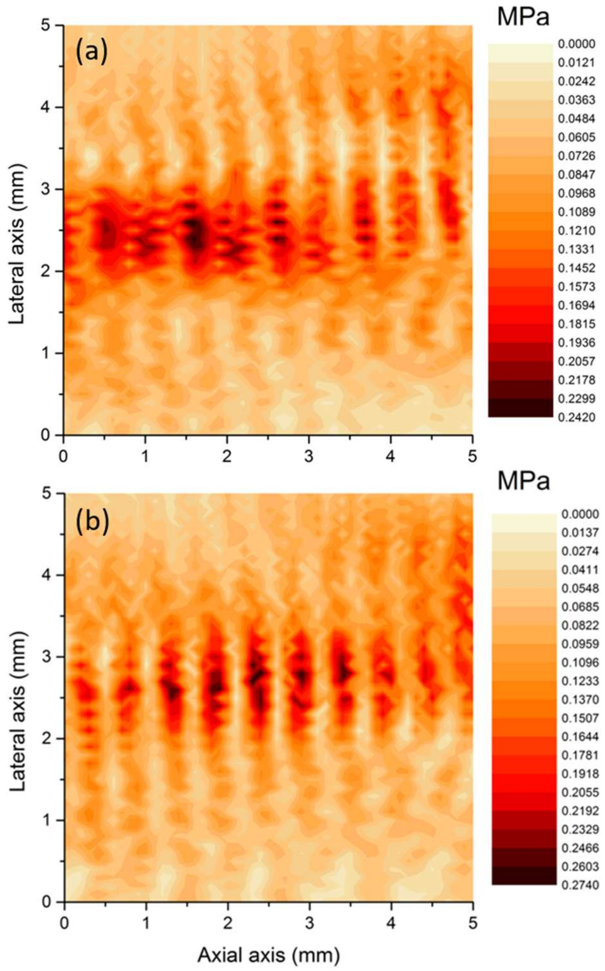


*Supplementary Figure 14: Pressure distributions at the focal zone of the confocal system with (a) one transducer and (b) both transducers driven by 5 Vpp continuous sinusoid at 1.495 MHz, respectively.*

In general, a particle in an ultrasonic standing wave is subjected to acoustic gradient forces, where the axial force can be expressed as:

$$F_a = -\frac{(\pi P^2 V \beta_w)}{2\lambda}\phi(\beta,\rho)\sin(2kx) \qquad (8\text{-}1)$$

$$\phi(\beta,\rho) = \frac{(5\rho_p - 2\rho_w)}{(2\rho_p + \rho_w)} - \frac{\beta_p}{\beta_m} \qquad (8\text{-}2)$$

where $P$, $V$, $\lambda$, $\beta$ and $\rho$ are the acoustic pressure amplitude, particle volume, wavelength, compressibility and density, respectively. The subscripts $p$ and $w$ denote particle and water respectively. The wave number, $k$, is defined as $k = 2\pi/\lambda$ and $x$ is the distance from the pressure node. The compressibility, $\beta$, is defined as $\beta = 1/(\rho c^2)$, where $c$ is the speed of sound. The acoustic contrast factor, $\emptyset$, determines whether the particle moves towards the pressure node ($\emptyset > 0$) or antinode ($\emptyset < 0$).

Neglecting spatial averaging and the 13% calibration uncertainty of the hydrophone, the measured maximum pressure amplitude, $P$ = 0.274 MPa, can be used to predict the acoustic forces on test particles of simplified geometry. Our results lead to peak axial forces, on test particles consisting of Ø 500-µm glass and polystyrene spheres, of $F_a$ = 5.72 µN and $F_a$ = 2.34 µN respectively, using the material properties listed in Supplemental Table 2 for water and Supplemental Table 4 for glass and polystyrene. Assuming a linear system, the pressure magnitude is proportional to the drive voltage and, as a consequence, the acoustic force should be proportional to the square of the excitation voltage amplitude applied to the transducers. Thus, an applied voltage V = 60 $V_{pp}$ is expected to yield a peak axial force $F_a$ = 823 µN on the Ø 500 µm glass sphere. This predicted force is approximately 165 times higher than what we were able to experimentally measure as the maximum lateral force on the glass sphere, yet that ratio is similar to that reported previously in the literature [5].

*Supplementary Table 4: Material properties of spherical particles*

| Material | Glass | Polystyrene |
|---|---|---|
| Density ($kg{\cdot}m^{-3}$) | 2300 | 1050 |
| Speed of sound ($m{\cdot}s^{-1}$) | 5200 | 2400 |

In comparison to optical trapping, it is generally considered impractical, if not impossible, to trap such large objects. For a single-beam 3D trap (optical tweezers) a “rule-of-thumb” for the force that can be generated is 1pN for every 10mW laser power applied[6]. Scaling this up to the 5µN required to trap the 500-µm diameter glass sphere results in a laser power of about 50 kW in a tightly focused beam. If instead we look at counter-propagating optical traps which are more favourable for trapping of larger objects, forces of 135pN can be achieved with 500mW laser power[7]. Again, for the example above, this would require 1.85 kW laser power in a ~ 1 mm diameter beam to achieve the required forces. This results in the highly impractical optical intensity at the trap location of approximately 600 $MW/cm^2$.

## Supplementary Note 9: Schlieren imaging of the acoustic fields

When the transducers are mounted on the sample chamber, the specific distance between the transducers and the acoustic properties of all materials involved affect the resonance frequency of the whole system. Hence our experimental determination of the optimal frequency to run the system was essential for creation of a successful acoustic trap.

Hydrophone scanning (as in Supplementary Figure 14) provides accurate measurement of the pressure and phase information in the acoustic field. However, this process is slow, requiring point-by-point raster scanning over a 3D volume, which can take hours. Alternatively, Schlieren imaging utilizes light to map the refractive index gradient in a uniform medium (integrated over the direction of propagation) and it has been proven to be a useful and much faster method to enable direct visualize of the acoustic fields, though without providing quantitative intensity measurements [8]. For this reason, we have used the Schlieren method to visualize our ultrasound pressure distribution, and furthermore, to determine, independently, the optimal frequency to drive the transducers.

Our Schlieren imaging setup was employed to visualize the acoustic pressure distribution in our light sheet microscopy sample chamber.

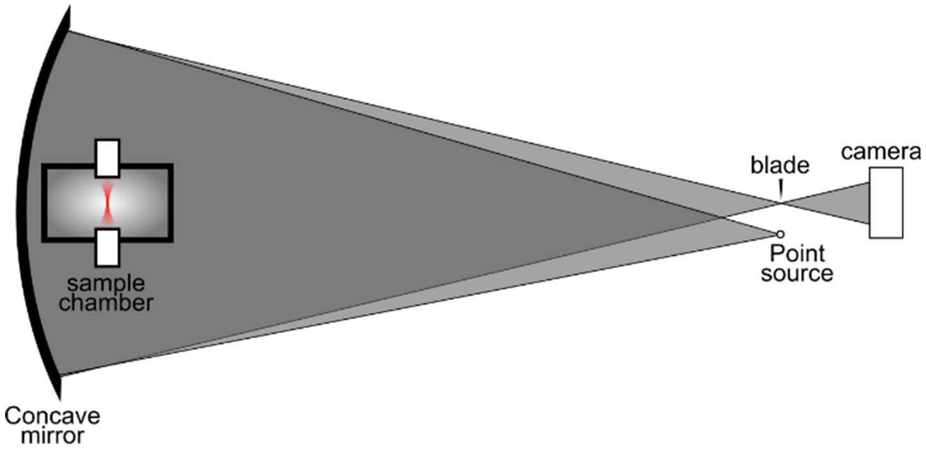


*Supplementary Figure 15: Schlieren setup. A point-like light source was constructed by housing a white LED in a metal tube, with an optical output aperture of ~ 1 mm. The concave spherical mirror (150 mm Dia. × 1500 mm focal length, Edmund optics) is mounted on a homemade rig, placing it twice its focal length from the point source. Light reflected from the concave mirror is half blocked at focal point by a razor blade, providing a phase contrast image of pressure gradients inside the sample chamber. A Digital Single Lens Reflex (DSLR) camera captures the images.*

Below are examples of our Schlieren images. A movie is available at **Supplementary Movie 8**. We manually swept the driving frequency from 1.3 MHz to 1.6 MHz so as to identify the optimal frequency for the system for operation.

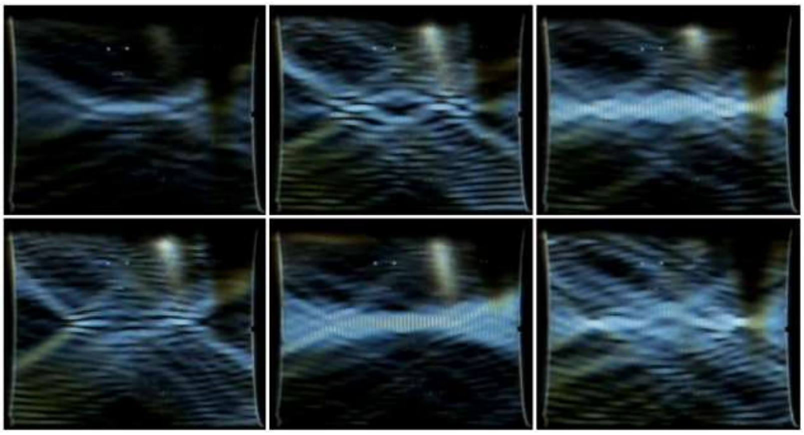

*Supplementary Figure* 16*: Examples of our Schlieren images at random frequencies. Two acoustic transducers were on left and right side of the image, generating ultrasound waves. The contrast in the image presents the acoustic pressure in the sample chamber. The vertical strips illustrate the acoustic standing waves.*

# Supplementary References


1. Schwerte, T. Development of the sympatho-vagal balance in the cardiovascular system in zebrafish (Danio rerio) characterized by power spectrum and classical signal analysis. *J. Exp. Biol.* **209,** 1093–1100 (2006).

2. De Luca, E. *et al.* ZebraBeat: a flexible platform for the analysis of the cardiac rate in zebrafish embryos. *Sci. Rep.* **4,** 4898 (2015).

3. Grewe, B. F., Voigt, F. F., van't Hoff, M. & Helmchen, F. Fast two-layer two-photon imaging of neuronal cell populations using an electrically tunable lens. *Biomed. Opt. Express* **2,** 2035 (2011).

4. Qiu, Y. *et al.* Screen-printed ultrasonic 2-D matrix array transducers for microparticle manipulation. *Ultrasonics* **62,** 136–146 (2015).

5. Woodside, S. M., Bowen, B. D. & Piret, J. M. Measurement of ultrasonic forces for particle–liquid separations. *AIChE J.* **43,** 1727–1736 (1997).

6. Neuman, K. C. & Block, S. M. Optical trapping. *Rev. Sci. Instrum.* **75,** 2787–2809 (2004).

7. Thalhammer, G., Steiger, R., Bernet, S. & Ritsch-Marte, M. Optical macro-tweezers: trapping of highly motile micro-organisms. *J. Opt.* **13,** 44024 (2011).

8. Neumann, T. & Ermert, H. Schlieren visualization of ultrasonic wave fields with high spatial resolution. *Ultrasonics* **44,** e1561–e1566 (2006).
