# Supplementary material for: Light sheet microscopy with acoustic sample confinement
Source: Nat Commun. 2019 Feb 8;10:669. doi: 10.1038/s41467-019-08514-5 (PMC6368588; doi:10.1038/s41467-019-08514-5)
Supplement: Supplementary file 10 — Description of Additional Supplementary Files [file 41467_2019_8514_MOESM10_ESM.docx]

Movie legends:

**Title:** Supplementary Video 1.
**Description:** Amphioxus embryo time-lapse video showing original three-dimensional morphology.

**Title:** Supplementary Video 2.
**Description:** Movement of the chorion in mid-gastrula stage Amphioxus embryos.

**Title:** Supplementary Video 3.
**Description:** Amphioxus embryos rotation speed increased with the embryo age.

**Title:** Supplementary Video 4.

**Description:** Amphioxus chorions spun in different directions when multiple embryos were trapped in the same acoustic gradient potential energy well.

**Title:** Supplementary Video 5.
**Description:** Vector video of zebrafish beating heart as in Figure 3.

**Title:** Supplementary Video 6.
**Description:** Strain video of zebrafish beating heart as in Figure 4.

**Title:** Supplementary Video 7.
**Description:** Section image of Ciona embryo in Figure. 2**b.**

**Title:** Supplementary Video 8.
**Description:** Schlieren video showing the acoustic field changes with ultrasound frequency.
